# Experimental and Clinical Applications of *Chamaecyparis obtusa* Extracts in Dry Eye Disease

**DOI:** 10.1155/2017/4523673

**Published:** 2017-12-26

**Authors:** Lian Cui, Hyo Seok Lee, Ying Li, Joo-Hee Choi, Je-Jung Yun, Ji Eun Jung, Won Choi, Kyung Chul Yoon

**Affiliations:** ^1^The Affiliated Eye Hospital, Wenzhou Medical University, Wenzhou, China; ^2^Department of Ophthalmology and Center for Creative Biomedical Scientists, Chonnam National University, Gwangju, Republic of Korea; ^3^Balgeun Eye Clinic 21, Gwangju, Republic of Korea; ^4^College of Veterinary Medicine and BK 21 PLUS Project Team, Chonnam National University, Gwangju, Republic of Korea; ^5^Business Supporting Team, Center for Nano Bio Research, Jangseong, Jeonnam, Republic of Korea; ^6^Jeonnam Forest Resource Research Institute, Naju, Jeonnam, Republic of Korea

## Abstract

**Purpose:**

To investigate the effects of *Chamaecyparis obtusa* (CO) on human corneal epithelial (HCE) cells, a murine experimental dry eye (EDE) model, and the efficacy of antioxidant eye mask in dry eye disease (DED) patients.

**Methods:**

0.001%, 0.01%, and 0.1% CO extracts were used to treat HCE cells, cell viability, and production of antioxidative enzymes, and reactive oxygen species (ROS) were assessed. Afterwards, CO extracts or balanced salt solution (BSS) was applied in EDE. Clinical and experimental parameters were measured at 7 days after treatment. In addition, DED patients were randomly assigned to wear either an eye mask containing CO extracts or a placebo. Clinical parameters were evaluated.

**Results:**

The viability of HCE cells and antioxidative enzyme expression significantly improved after treatment with 0.1% CO extracts. Mice treated with 0.1% CO extracts showed significant improvement in clinical parameters. During the trial, the clinical parameters significantly improved in the treatment group at 4 weeks after application.

**Conclusions:**

0.1% CO extracts could promote the expression of antioxidative proteins and ROS production. In addition, an eye mask containing CO extracts could improve DED clinical parameters. These suggest that CO extracts may be useful as an adjunctive option for the DED treatment.

## 1. Introduction

Dry eye disease is one of the most common ophthalmic pathologies that results in symptoms of discomfort, visual disturbance, and tear instability with potential damage to the ocular surface [1]. Moreover, dry eye disease is a chronic ocular disorder affecting about 14.5% of the world's population including 17.9% of women and 10.5% of men, and the prevalence continues to rise [2]. The pathology of this condition involves inflammation of the ocular surface, in which T cells are highly involved [3, 4]. However, the pathogenesis of the disease has not yet been fully elucidated.

Recently, it was recognized that oxidative stress plays a notable role in dry eye disease [5–8]. Oxidative stress is caused by an imbalance between the production of reactive oxygen species (ROS) and the ability of biological systems' defense mechanisms necessary to eliminate the stress [9]. Excessive oxidative stress is associated with ocular surface epithelial damage, as well as with a decrease in the secretory function of the lacrimal gland [10]. The damaged epithelial cells then release cytokines and cause ocular surface inflammation, resulting in dry eye disease [11].

A variety of antioxidative treatments, such as omega-3 essential fatty acids, blueberry component, xanthan gum, oral sea buckthorn oil, and green tea polyphenols, has been shown to prevent or treat dry eye disease [5, 8, 12–14]. In a previous study, we demonstrated the efficacy of mixed medicinal plants with antioxidant and anti-inflammatory properties against oxidative stress induced by irradiation from a short wavelength light-emitting diode or desiccating stress in human corneal epithelial (HCE) cells and in a mouse model of experimental dry eye (EDE) [6, 15]. In addition, we also showed that glasses containing extracts of medicinal plants with antioxidative property could improve subjective and objective parameters in patients with dry eye disease [16].

*Chamaecyparis obtusa* (CO) is a tropical tree species found in Japan and in the southern regions of South Korea; the species has various biological activities, including cytotoxic, antibacterial, antifungal, antioxidative, antiapoptotic, and anti-inflammatory effects [17–24]. Furthermore, although the biological activity of essential oil from CO is not fully understood, the oil dose contains several types of terpenes (sabinene, limonene, bornyl acetate, borneol, a-terpineol, and elemol), all of which have been shown to exert antioxidative and anti-inflammatory effects [23, 25].

Based on these effects, we hypothesized that CO extracts have potential benefits in the treatment of dry eye disease that are similar to their protective effects against oxidative stress and inflammation in other organs and tissues. In the present study, we investigated the role of these CO extracts on oxidative stress and inflammatory markers in HCE cells and on clinical parameters in a mouse model of EDE and in patients with dry eye disease.

## 2. Materials and Methods

### 2.1. Preparation of the Leaf Extract

Leaves of CO were collected at Jangseong Province in South Korea. After harvesting, the fresh leaves were washed with water and dried in a drying chamber using forced air under the temperature of 40°C for 10 days after which dry matter with a water content of less than 5% remained. The dry leaves were then milled to a size of 0.5 mm using a pin-type mill.

The leaf extract was processed with supercritical CO_2_ extraction system (ISA-SCFE system, Ilshin Company, Daejeon, South Korea) in the Nano Bio Research Center at Jangseong Province. Pure CO_2_ was applied using a syringe pump. Each ground leaf, weighing between 100 g and 125 g, was placed in a separate chamber and extracted supercritically; CO_2_ was used as the main extraction gas, C_2_H_3_OH was used as the cosolvent, and the procedure was performed under a pressure of 200 bars [26, 27]. During the extraction process, the pressure, temperature, and CO_2_ flow rate were controlled through adjustment of the regulating valves. Each separation vessel was set at a temperature of 40°C, a pressure of 200 bar, a CO_2_ flow rate of 60 mL/min, and a cosolvent flow rate of 3 mL/min. The parameters were optimized using a pretest and were determined based on extraction efficacy and operational performance. The pressure and temperature were optimized using an experimental design because they are critical for extraction. After 2 hours of extraction time, the extraction vessel was depressurized and the extracts were collected. The characteristics of CO extracts were clear yellow liquid with a density of 0.954 g/mL. It was stored in a clean vial at −20°C until use.

Diluted solutions were prepared using either phosphate buffer saline (PBS, for the *in vitro* experiments) or a balanced salt solution (BSS, for *in vivo* experiments and the clinical trial) at concentrations of 0.001%, 0.01%, and 0.1%.

### 2.2. Cell Culture and Viability

A human SV-40 immortalized corneal epithelial cell line (CRL-11135, HCE-2; ATCC, Manassas, VA, USA) at passage 28 was cultured at 37°C in a humidified incubator containing 5% CO_2_. They were maintained in Dulbecco's Modified Eagle's medium (Cascade Biologics, Portland, OR, USA). The medium was replaced every other day and supplemented with human corneal growth supplement (Cascade Biologics) with 100 U/mL of penicillin and 100 *μ*g/mL of streptomycin [28].

Cell viability was measured using the EZ-Cytox assay kit (Daeillab Service, Seoul, South Korea), which is based on the cleavage of tetrazolium salt into water-soluble formazan by succinate-tetrazolium reductase [29]. Approximately, the HCE cells (2 × 10^5^ cells/well) were seeded in a 96-well plate 24 hours prior to treatment and exposed to different concentrations of CO extracts (0.001%, 0.01%, and 0.1%) for another 1 hour. In addition, the cells (in 96-well plates) were added to 10 *μ*L of EZ-Cytox reagent in each well, and the plate was reincubated for 3 hours in a CO_2_ incubator. Absorbance was detected at a 570 nm wavelength, and an EL × 808 microplate spectrophotometer reader (BioTek, Winooski, VT, USA) was used. Hydrogen peroxide (H_2_O_2_; Fisher Scientific, Leicestershire, UK) was used as a positive control to assess the effect of the CO extracts on cell viability after oxidative stress. The cells were pretreated using CO extracts at the indicated concentrations for 1 hour before exposure to H_2_O_2_ (200 *μ*M). Exposure to H_2_O_2_ lasted for 30 min. Thereafter, cell viability was measured using the EZ-Cytox assay. The CO extract-pretreated and H_2_O_2_-treated cells were used in other *in vitro* analyses, and each assay was performed in triplicate.

### 2.3. Measurement of Intracellular ROS Production

Intracellular ROS production was monitored using a dihydroethidium (DHE) assay kit (D-23107; Life Technologies™, Darmstadt, Germany). After 12 hours of treatment in a 96-well plate, the cells were washed three times using PBS and incubated with 5 *μ*M DHE at 37°C for 30 minutes in the dark; they were then washed again three times using PBS. Cellular fluorescence was quantified using fluorescence microscopy (Eclipse TE-300; Nikon, Melville, NY, USA) at an excitation setting of 518 nm and an emission setting of 605 nm [30]. The DHE fluorescence intensities were quantified using Image J software (version 1.45; NIH, Bethesda, MD, USA.) and expressed as percentages normalized to a control.

### 2.4. Protein Extraction and Western Blot Analysis

Cells were lysed in RIPA buffer (number 9806; Cell Signaling Technology, Danvers, MA, USA) containing a protease inhibitor cocktail (Sigma-Aldrich Corp., St. Louis, MO, USA) and phosphatase inhibitor cocktail I + II (Sigma-Aldrich Corp.). An NE-PER nuclear and cytoplasmic extraction kit (number 78835; Thermo Fisher Scientific, San Jose, CA, USA) was used to extract nuclear proteins.

The expression levels of cleaved peroxiredoxin- (Prx-) 1, heme oxygenase- (HO-) 1, catalase (CAT), and cyclooxygenase- (COX-) 2 were examined by Western blot analysis. Protein concentrations were determined using the Bradford procedure. Proteins from each variable were separated by sodium dodecyl sulfate polyacrylamide gel electrophoresis and transferred to nitrocellulose membranes. The membranes were incubated at room temperature in a blocking solution composed of 5% skimmed milk for 1 hour. They were then incubated with the primary antibodies overnight at 4°C. The samples were washed three times using Tris-buffered saline-Tween-20 (10 mM Tris-HCl, pH 7.6, 150 mM NaCl, 0.1% Tween-20); immunoreactive bands were then developed by enhanced chemiluminescence using EZ-Western Lumi Pico Western Blotting Detection Reagent (Daeil Lab Service) on a luminescent image analyzer (Image Quant LAS4000 mini, GE Healthcare Life Sciences, Little Chalfont, Buckinghamshire, United Kingdom).

### 2.5. Mouse Model of Dry Eye and Experimental Procedure

This research protocol was approved by the Chonnam National University Medical School Research Institutional Animal Care and Use Committee (CNU IACUC-H-2015-11). All *in vivo* studies adhered to the Association for Research in Vision and Ophthalmology Statement for the Use of Animals in Ophthalmic and Vision Research.

Female C57BL/6 mice aged 6 to 8 weeks were used in these experiments. The mouse model of EDE was induced by subcutaneous injection of 0.5 mg/0.2 mL scopolamine hydrobromide (Sigma-Aldrich Corp) four times a day (8 AM, 11 AM, 14 PM, and 17 PM) with exposure to an air draft and 30% ambient humidity [3, 31]. The mice were randomly assigned to five to six groups based on the topical treatment administered as follows: (1) untreated control (UT), not exposed to desiccating stress or treated topically; (2) EDE control mice that received no eye drops; (3) EDE mice treated with BSS (Alcon, Fort Worth, TX, USA); (4) EDE mice treated with 0.001% CO extracts; (5) EDE mice treated with 0.01% CO extract; and (6) EDE mice treated with 0.1% CO extract. All treatment groups received 3 *μ*L of eye drops 3 times a day (8 AM, 12 PM, and 5 PM). The following clinical parameters including tear volume, tear film break-up time (TBUT), and corneal fluorescein staining scores were measured at 7 days after treatment. Mice were euthanized 7 days after the induction of desiccating stress, and multiplex immunobead assay and conjunctival ROS level analysis were performed.

### 2.6. Measurement of Tear Volume

Tear volume was measured using phenol red-impregnated cotton threads (Zone-Quick; Showa Yakuhin Kako Co., Tokyo, Japan) 3 hours after the last scopolamine injection, as previously described [6, 32]. The threads were placed in the lateral canthus for 20 seconds. When the thread was wet due to tears, it turned red. The tear volume, expressed in micrometers of red thread, was measured using a microscope (SMZ 1500; Nikon, Melville, NY, USA). A standard curve was derived to convert distance into a volume with a known uptake volume of basic stock solution (1500 mL of 0.9% saline combined with 5 mL of 5 M NaOH) over 20 s.

### 2.7. Evaluation of Tear Film Break-Up Time and Corneal Fluorescein Staining

Fluorescein (1%; 1 *μ*L) was instilled into the inferior conjunctival sac; after three blinks, the TBUT was recorded in seconds using slit lamp biomicroscopy (BQ-900; Haag-Streit, Bern, Switzerland) under a cobalt blue light. Ninety seconds later, punctate staining on the corneal surface was measured in a masked fashion. Each cornea was divided into four quadrants, each of which was scored individually. The intensity of corneal fluorescein staining score was calculated using a 4-point scale: 0, absent; 1, superficial stippling micropunctate staining with <30 spots; 2, punctate staining with >30 spots, but no diffuse staining; 3, severe diffuse staining, but no positive plaque/patch; and 4, positive fluorescein plaque/patch [3]. The scores of the four areas were summed to generate a final grade, ranging from 0 to 16.

### 2.8. Multiplex Immunobead Assay

A multiplex immunobead assay (Luminex 200; Luminex Corp., Austin, TX, USA) was used to measure the concentrations of interleukin- (IL-) 17, interferon- (IFN-) *γ*, tumor necrosis factor- (TNF-) *α*, and IFN-*γ*-induced protein- (IP-) 10 in the conjunctiva, as previously described [28, 31]. The tissues were collected and pooled in lysis buffer containing protease inhibitors for 30 minutes. The cell extracts were then centrifuged at 14,000*g* at 4°C for 15 minutes, and the supernatants were stored at −70°C until required. The total protein concentration of the supernatants was determined, and 25 *μ*L of each sample was pipetted into assay plate wells. The wells contained the appropriate cytokine and chemokine bead mixture (Millipore, Billerica, MA, USA), as well as mouse monoclonal antibodies specific for the mixture. Next, the supernatant was washed three times and a biotinylated secondary antibody mixture was applied for 30 minutes in the dark at room temperature. The reactions were detected using an analysis system (xPONENT, Austin, TX, USA) after streptavidin-phycoerythrin had been added. The concentrations of tissue cytokines and chemokines in the samples were calculated through comparison with standard curves of known concentrations.

### 2.9. Measurement of Conjunctival ROS Production

The level of intracellular ROS was measured using a DCF-DA assay according to the manufacturer's protocol, as previously described [6]. The conjunctiva was surgically excised and washed using PBS and 10 *μ*M DCF-DA (Molecular Probes, Eugene, OR, USA); it was then incubated for 30 minutes at 37°C. The cell pellet was analyzed using a FACScalibur flow cytometer (BD Biosciences, San Jose, CA, USA) at excitation and emission wavelengths of 488 and 530 nm, respectively. The relative changes in DCF-DA fluorescence were expressed as fold increase compared to that in the control tissue (conjunctivas harvested from mice that had not been exposed to desiccant stress or topical treatment).

### 2.10. Patients and Study Design

Informed consent was obtained from all patients in accordance with the Declaration of Helsinki, and the study protocol was approved by the Institutional Review Board of Chonnam National University Hospital (CNUH-2015-232). The International Standard Randomized Controlled Trial Number (ISRCTN) is ISRCTN10704205.

Patients aged between 20 and 60 years with dry eye disease were recruited. The eligibility criteria for patients entering the study were as follows: (1) one or more dry eye-related ocular symptoms (>3 months) such as dryness, irritation, and burning sensations; (2) ocular surface disease index (OSDI) score > 15; (3) TBUT < 10 s; and (4) Schirmer's test (with application of local anesthetic) value < 10 mm for 5 minutes [16]. Pregnant women were excluded from the study, as were patients with (1) active eye and periocular skin inflammation, (2) vitamin A deficiency, (3) a history of ocular surgery < 3 months before the study, (4) a history of wearing contact lenses, (5) a history of active treatment for dry eye (punctual occlusion or use of anti-inflammatory eye drops < 1 month before the study), and (6) systemic conditions or medication that may have caused dry eye.

This prospective study included 50 patients who were recruited at Chonnam National University Hospital in January 2016. Patients were randomly assigned to either the treatment or the placebo group. The treatment group received a sleeping eye mask composed of an external supporting frame with masks that contain CO extracts at the inside of the frontal part of the frame. Patients in the placebo group were given an identical frame with masks that did not contain the CO extracts. During the study, the participants used the same mask for 7 hours when they slept at night. The examinations were performed at baseline and 2 and 4 weeks after eye mask application. At each visit, each subject underwent detailed ocular examinations including best-corrected visual acuity, slit lamp biomicroscopy, OSDI, TBUT, and Schirmer's test. Participants were tested between 10 AM and 11 AM at each visit. For safety reasons, all patients were also questioned regarding any ocular symptoms related to the eye mask at all visits.

Subjective dry eye symptoms were assessed using the OSDI score (range: 0–100) [33]. The following objective clinical parameters were evaluated: TBUT (seconds) and Schirmer's test (with anesthesia) [34]. These tests were performed by a single investigator (K.C.Y.), as described previously. TBUT was assessed using slit lamp biomicroscopy (BQ-900; Haag-Streit, Bern, Switzerland), and a cobalt blue filter 2 minutes after 2 *μ*L of 0.5% fluorescein had been instilled. This test was repeated three times, and the average value was calculated. Schirmer's test was performed 5 minutes after instillation of a 2 *μ*L drop of 0.5% fluorescein, and 0.4% oxybuprocaine hydrochloride had been instilled in the conjunctival sac. A standard Schirmer's test strip was then placed in the lateral canthus for another 5 minutes, and the patient closed their eyes. The length of wetting on the strip was measured using a millimeter scale. All participants included in the analysis completed the clinical trial without dropout participants. Compliance was monitored by the examiner at the time of each visit through the wearing time diary.

### 2.11. Statistical Analysis

The data are presented as mean ± standard deviations. The statistical package for the social sciences (SPSS) software (version 18.0; SPSS, Chicago, IL, USA) was used for statistical analyses. A Kruskal–Wallis test with Bonferroni post hoc analysis was used to compare cell viability, cytokine and chemokine levels, and DCF-DA value differences among the groups. The normal distribution of the data was verified using the Kolmogorov–Smirnov test. Statistical differences in tear volume, TBUT, and corneal fluorescein staining among the groups were determined using one-way analysis of variance tests followed by Dunnett's post hoc tests (sphericity assumptions were evaluated with Mauchly's test, and in the case of violation, the data were adjusted with an Epsilon Greenhouse–Geisser statistic). In the clinical study, to ensure similarity between the treatment and placebo groups at baseline, an independent *t*-test was performed. Changes in the outcome measures over the follow-up period were assessed using a repeated measure analysis of variance, followed by Bonferroni's post hoc test. A *P* value < 0.05 was considered statistically significant.

## 3. Results

### 3.1. CO Extracts Attenuated H_2_O_2_-Induced Cell Loss

**Figure 1:**
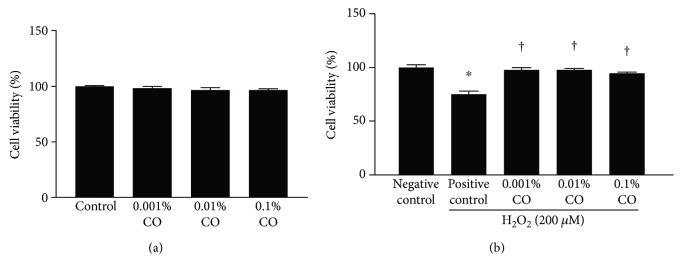
Effect of pretreatment with *Chamaecyparis obtusa* (CO) extracts on the viability of human corneal epithelial (HCE) cells. (a) The effect of CO extracts (0.001%, 0.01%, and 0.1% concentrations) on the viability of HCE cells is shown. (b) The effect of CO extracts (0.001%, 0.01%, and 0.1% concentrations) on HCE cells exposed to 200 *μ*M hydrogen peroxide (H_2_O_2_). The results are shown as a percentage relative to the control samples. ^∗^*P* < 0.01 compared with the control cells. ^†^*P* < 0.01 compared with the cells exposed to H_2_O_2_.

As seen in Figure 1(a), CO extracts at 0.001%, 0.01%, and 0.1% concentrations did not have any significant effect on the viability of HCE cells. The cell viability significantly decreased in the positive control group after exposure to 200 *μ*M H_2_O_2_ (75.06% ± 2.98%, *P* < 0.01 versus control). The viabilities of HCE cells in different CO pretreatment groups (0.001%, 0.01%, and 0.1%) were 97.72% ± 2.32%, 97.85% ± 1.25%, and 94.70% ± 1.03%, respectively (all *P* > 0.05 versus control and all *P* < 0.01 versus H_2_O_2_ control).

### 3.2. CO Extracts Prevented H_2_O_2_-Induced Oxidative Stress in HCE Cells

**Figure 2:**
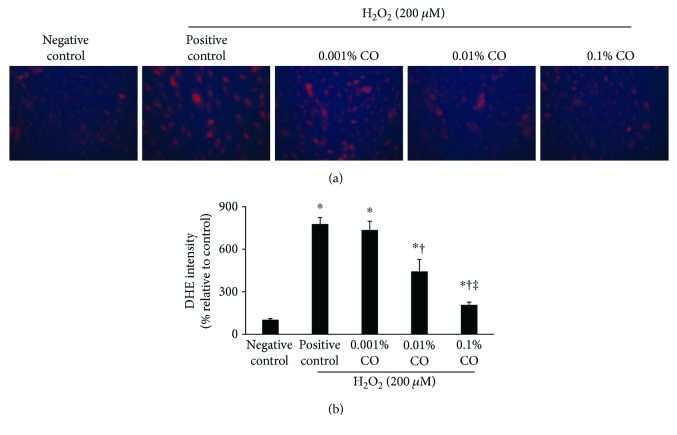
Dihydroethidium (DHE) staining and the subsequent confocal fluorescence microscopy observations in hydrogen peroxide- (200 *μ*M) treated human corneal epithelial cells with or without pretreatment with *Chamaecyparis obtusa* (CO) extracts (0.001%, 0.01%, and 0.1% concentrations). (a) One representative image selected from three individual experiments is shown. (b) Relative fluorescence intensity (expressed as percent normalized to negative control) analysis is shown. ^∗^*P* < 0.05 compared with the control cells. ^†^*P* < 0.05 compared with the H_2_O_2_-exposed cells. ^‡^*P* < 0.05 compared with the 0.001% CO extract-pretreated cells.

Figure 2(a) illustrates magnified representative images of DHE staining, as well as an analysis of relative fluorescence intensity. A significant increase in DHE fluorescence was seen after exposure to 200 *μ*M H_2_O_2_. However, pretreatment with CO extracts significantly reduced the DHE intensity in a concentration-dependent manner (0.001% CO: *P* = 0.96 versus positive control; 0.01% CO: *P* = 0.04 versus positive control; 0.1% CO: *P* < 0.01 versus positive control; and *P* = 0.01 versus 0.001% CO).

### 3.3. Effect of CO Extracts on Antioxidative and Anti-Inflammatory Markers

**Figure 3:**
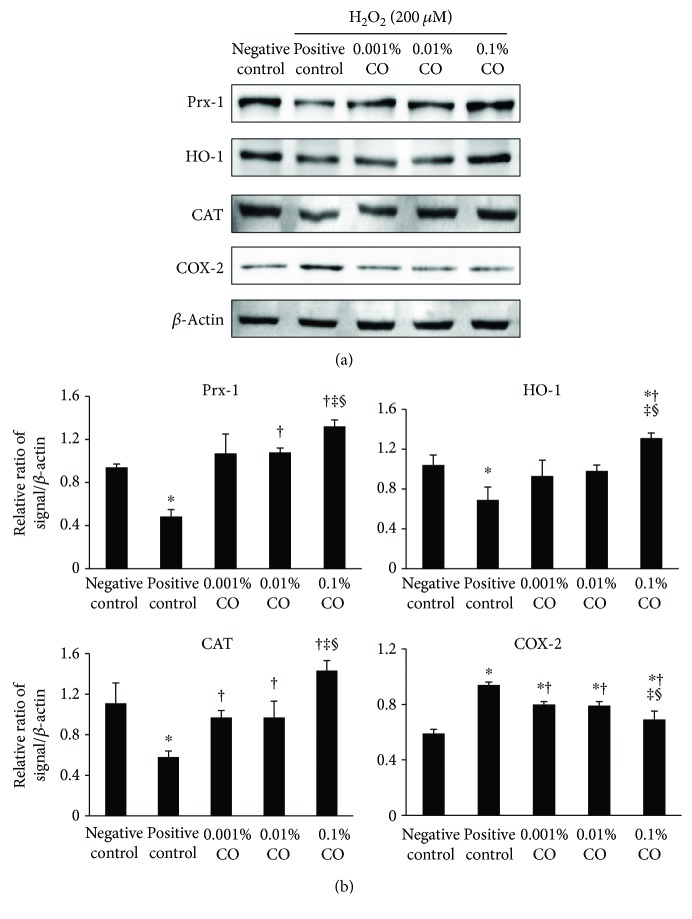
Western blot analyses to evaluate the effect of *Chamaecyparis obtusa* (CO) extracts on the expression of peroxiredoxin- (Prx-) 1, heme oxgenase- (HO-) 1, catalase (CAT), and cyclooxygenase-2 (COX-2) in the presence or absence of hydrogen peroxide (negative control), H_2_O_2_ control (positive control), and 0.001%, 0.01%, and 0.1% CO extract groups. *β*-Actin was used as an internal control. (a) One representative data set obtained from among three individual experiments is shown. (b) Western blot band intensity analyses (relative ratio of signal/*β*-actin) are shown. ^∗^*P* < 0.05 compared with the control group. ^†^*P* < 0.05 compared with the H_2_O_2_ control group. ^‡^*P* < 0.05 compared with the 0.001% CO extracts group. ^§^*P* < 0.05 compared with the 0.01% CO extract group.

As shown in Figure 3, the levels of Prx-1, HO-1, and CAT were significantly decreased after exposure to H_2_O_2_. However, they increased in a dose-dependent manner after pretreatment using CO extracts. The level of COX-2 increased after exposure to H_2_O_2_ and was significantly lower in the 0.1% CO extract group than in the H_2_O_2_ control, the 0.001%, and the 0.01% CO extract groups. In addition, the 0.001% and 0.01% CO groups differed significantly from the positive control group in this regard.

### 3.4. Tear Volume

**Figure 4:**
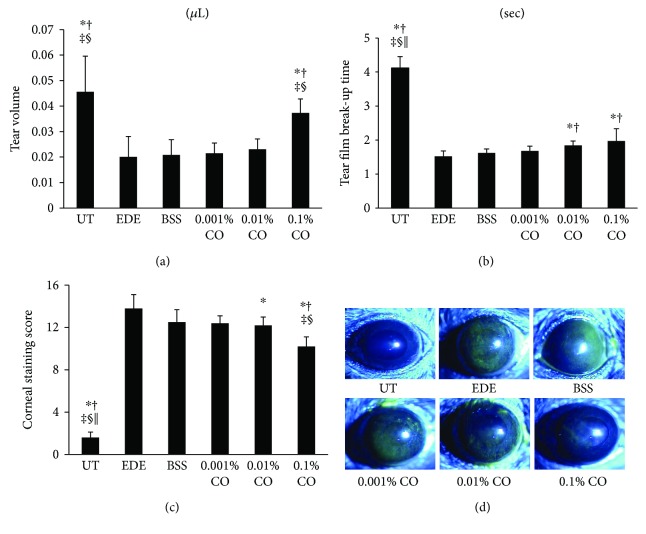
Mean tear volumes (a), tear film break-up time (b), corneal fluorescein staining scores (c), and representative figures (d) of the untreated (UT) control, experimental dry eye (EDE), balanced salt solution (BSS), and 0.001%, 0.01%, and 0.1% *Chamaecyparis obtusa* (CO) extract groups 7 days after desiccant stress. ^∗^*P* < 0.05 compared with the EDE group. ^†^*P* < 0.05 compared with the BSS group. ^‡^*P* < 0.05 compared with the 0.001% CO extract group. ^§^*P* < 0.05 compared with the 0.01% CO extract group. ^||^*P* < 0.05 compared with the 0.1% CO extract group.

There were no statistically significant differences in tear volumes among the groups at baseline (data not shown). Seven days after the desiccating stress was applied; the mean tear volumes of the five groups were 0.046 ± 0.014 *μ*L, 0.021 ± 0.006 *μ*L, 0.022 ± 0.004 *μ*L, 0.023 ± 0.004 *μ*L, and 0.037 ± 0.006 *μ*L, respectively, in the UT, BSS, 0.001%, 0.01%, and 0.1% CO groups. Tear volume in the 0.1% CO treatment group was significantly greater than those in the other groups (all *P* < 0.01; Figure 4(a)).

### 3.5. Tear Film BUT

There were no statistically significant differences in TBUT among the groups at baseline (data not shown). Seven days after desiccating stress had been applied, the TBUT was found to be 1.52 ± 0.16 s and 1.62 ± 0.12 s in the EDE and BSS groups, respectively; it was significantly shorter than that in the UT group (4.12 ± 0.33 s, *P* < 0.01; Figure 4(b)). The 0.01% and 0.1% CO extract groups (1.84 ± 0.13 s and 1.97 ± 0.36 s, resp.) had significantly greater TBUT values than the EDE (*P* < 0.01 and *P* = 0.03, resp.) and BSS (*P* < 0.01 and *P* = 0.01, resp.) groups. However, the 0.001% CO extract group did not differ significantly from the EDE and BSS groups in terms of TBUT. (1.68 ± 0.14 s; *P* = 0.27 and *P* = 0.98, resp.).

### 3.6. Corneal Fluorescein Staining

At baseline, the mean corneal fluorescein staining scores showed no significant differences among the groups (data not shown). On day 7, the corneal fluorescein staining score in the 0.001% CO group was 12.40 ± 0.70; it showed no significant difference compared with the EDE (13.80 ± 1.32, *P* = 0.09) and BSS groups (12.50 ± 1.18, *P* = 0.99; Figure 4(c)). The 0.01% CO extract group showed a significantly lower corneal fluorescein score than the EDE group (12.20 ± 0.79, *P* = 0.04), and the 0.1% CO treatment group showed a significantly lower corneal fluorescein staining score than the EDE, BSS, 0.001%, and 0.01% CO groups (10.20 ± 0.92, all *P* < 0.01).

### 3.7. Inflammatory Cytokine and Chemokine Levels in the Conjunctival Tissue

**Figure 5:**
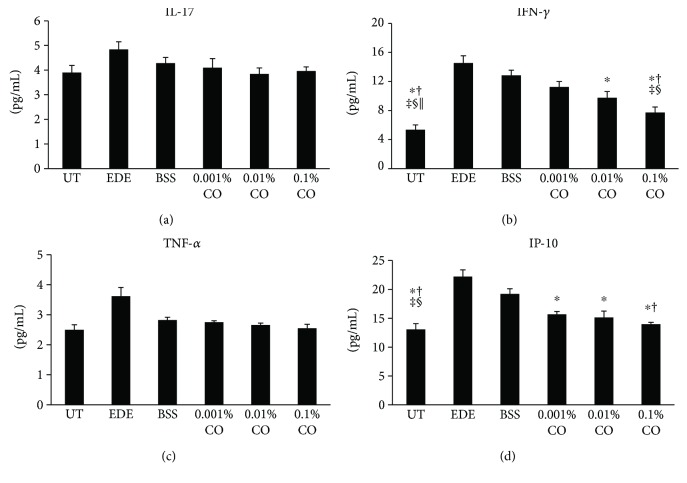
Concentrations of interleukin- (IL-) 17 (a), interferon- (IFN-) *γ* (b), tumor necrosis factor- (TNF-) *α* (c), and IFN-*γ*-inducible protein- (IP-) 10 (d), in the conjunctiva of the untreated (UT) control, experimental dry eye (EDE), balanced salt solution (BSS), and 0.001%, 0.01%, and 0.1% *Chamaecyparis obtusa* (CO) extract groups. ^∗^*P* < 0.05 compared with the EDE group. ^†^*P* < 0.05 compared with the BSS group. ^‡^*P* < 0.05 compared with the 0.001% CO extract group. ^§^*P* < 0.05 compared with the 0.01% CO extract group. ^||^*P* < 0.05 compared with the 0.1% CO extract group.

The inflammatory cytokine and chemokine levels in conjunctival tissues are shown in Figure 5. The mean concentration of IFN-*γ* was 7.73 ± 0.75 pg/mL in the 0.1% CO group. This was significantly lower than the concentrations in the EDE, BSS, and 0.001% CO groups (14.54 ± 1.00 pg/mL, 12.84 ± 0.72 pg/mL, and 11.24 ± 0.74 pg/mL and *P* < 0.01, *P* < 0.01, and *P* = 0.03, resp.). The 0.01% CO group showed significantly lower levels of IFN-*γ* than the EDE group (9.76 ± 0.86 pg/mL, *P* = 0.02); however, the levels were not lower than those in any of the other groups.

The mean levels of IP-10 were 15.66 ± 0.51 pg/mL, 15.14 ± 1.12 pg/mL, and 13.98 ± 0.32 pg/mL in the 0.001%, 0.01%, and 0.1% CO groups, respectively, after 7 days of desiccating stress. The 0.1% CO group showed significantly lower levels than the EDE and BSS groups (22.23 ± 1.15 pg/mL and 19.21 ± 0.89 pg/mL, *P* = 0.02 in both case). The 0.001% and 0.01% CO groups showed significantly lower levels than the EDE group (*P* = 0.02, *P* < 0.01).

The concentrations of IL-17 and TNF-*α* were lower in the CO treatment groups than in the EDE group, but there were no statistically significant differences.

### 3.8. Measurement of ROS Level in the Conjunctival Tissue

**Figure 6:**
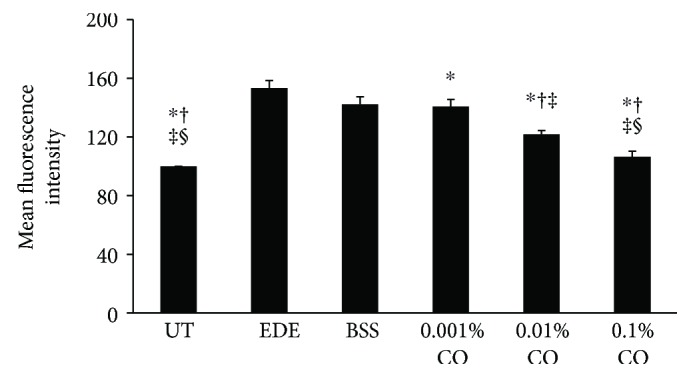
Mean conjunctival fluorescein intensity showing the reactive oxygen species (ROS) levels in the untreated (UT) control, experimental dry eye (EDE), balanced salt solution (BSS), and 0.001%, 0.01%, and 0.1% *Chamaecyparis obtusa* (CO) extracts groups. ^∗^*P* < 0.05 compared with the EDE group. ^†^*P* < 0.05 compared with the BSS group. ^‡^*P* < 0.05 compared with the 0.001% CO extract group. ^§^*P* < 0.05 compared with the 0.01% CO extract group.

The fluorescence intensities of DCF-DA in representative samples from the UT, EDE, BSS, 0.001%, 0.01%, and 0.1% CO groups are shown in Figure 6. The respective ROS levels were 100 ± 0.00, 153.33 ± 5.09, 142.27 ± 5.10, 140.63 ± 5.00, 121.80 ± 2.56, and 106.50 ± 3.68. Treatment with 0.001%, 0.01%, and 0.1% CO extracts resulted in a significantly greater decrease in ROS level when compared to that in the EDE group (*P* = 0.04, *P* < 0.01, and *P* < 0.01, resp.). Furthermore, treatment with 0.01% and 0.1% CO extracts resulted in a significantly greater decrease in ROS level when compared to those in the BSS and 0.001% CO groups (all *P* < 0.01).

### 3.9. Symptoms, Tear Film, and Ocular Surface Parameters in Patients

No statistically significant differences were detected between the two groups with regard to the baseline characteristics (age, gender, visual acuity OSDI score, TBUT, Schirmer's test score, and slit lamp findings; data not shown).

In the placebo group, there was no significant improvement in OSDI scores during the study; however, in the treatment group, the OSDI score after 4 weeks (*P* < 0.01) had improved significantly over that at baseline and after 2 weeks (Table 1) In addition, intergroup comparison revealed that the OSDI score was significantly lower in the treatment group after 4 weeks of follow-up (*P* < 0.01).

Table 2 shows the TBUT values of both groups during the follow-up period. In the treatment group, the TBUT after 4 weeks of treatment was significantly better than that in the placebo group (*P* = 0.02). Furthermore, the treatment group had shown significant improvements over baseline after 4 weeks (*P* = 0.01).

As shown in Table 3, Schirmer's test showed no significant changes observed in either the treatment or the placebo groups. In addition, no significant differences were observed between the two groups during follow-up.

## 4. Discussion

The protective properties of CO have been recognized to exert antigastropathic, anti-inflammatory, and antioxidative activity [35]. However, little is known about the bioactivity and potential clinical implications of CO on the health of the human eye. The present study indicated that CO might have beneficial effects on eye and ocular surface disease. In the present study, the cellular viability has decreased subtly in the 0.1% group. We thought that it may be due to mild toxic reaction at a high concentration similar to general substances. However, it did not show a statistically significant change of the cellular viability in the present study. We also showed that CO reduced ROS generation and rebalanced homeostasis between oxygenases and antioxidative enzymes in HCE cells exposed to oxidative stress and that it decreased inflammation and ROS levels in a murine dry eye model. The mechanisms of ROS reduction by CO are as follows: phenolic compounds have been reported to be one of the major constituents of CO [36]. The antioxidant properties of phenolic compounds are related to their structural characteristics, which are known to be due to their role as direct ROS scavenging, reducing, and chain breaking antioxidant mechanisms [37].

Extensive researches during the past decade have demonstrated that oxidative stress played an important role in ocular surface disease, including dry eye [9, 38]. For example, *in vitro* studies have revealed that lipid and mitochondrial oxidative damage might cause inflammation in HCE cells [15, 38]. In addition, *in vivo* lipid oxidative injury has been observed in patients with dry eye, as well as in those with Sjögren syndrome [39]. Higher oxidative stress may also explain the higher incidence and more severe dry eye in elderly patients [40]. Our previous study established that a natural mixture of plant ethyl alcohol extracts has antioxidant effects, as well as protective effects against desiccating stress on the ocular surface [6].

In the *in vitro* part of the present study, CO extracts increased the expression of Prx-1, CAT, and HO-1 in a dose-dependent manner. The Prx is a new family of antioxidative proteins that inactivate ROS, thereby protecting tissues from oxidative stress [41]. CAT is an effective protector from oxidative stress, and HO-1 has a cytoprotective effect under various pathophysiological stimulating conditions, such as oxidative stress [42]. As a very important enzyme in protecting the cell from oxidative damage, CAT showed increased expression after pretreatment with CO extracts. The enzyme COX, which comprises two major isoforms, COX-1 and COX-2, catalyzes the metabolism of arachidonic acid to prostaglandins. Upregulation of COX-2 is a common feature of inflammation due to oxidative stress. Relatedly, COX-2 is stimulated by lipid peroxidation, and it mediates oxidative DNA damage [9, 38]. In the present study, the expression of COX-2 in the CO groups significantly decreased when compared with that in the positive control group. These antioxidative protein expressions may increase in consequence of cellular viability recovered by CO extracts. In addition, DHE analysis in the present study showed that CO extracts could reduce ROS production. These findings demonstrate that CO extracts may protect HCE cells from oxidative damage by reintroducing the balance between oxygenase and antioxidative enzymes, thus reducing ROS levels.

Several antioxidants have shown beneficial effects in murine models of dry eye, as well as in dry eye patients [4–6, 16]. In the aspects of the results obtained from our prior studies, oxidative stress played a notable role in the development of the ocular surface inflammation. Therefore, we hypothesized that antioxidative topical extracts could be beneficial in treating dry eye disease. Thus prompted, we evaluated the clinical efficacy of CO extracts on a murine dry eye model.

Despite continuous exposure to desiccating stress and rigorous anticholinergic treatment, various clinical parameters including tear volume, TBUT, and corneal fluorescein staining score showed clinical improvements on day 7 in the 0.1% CO group compared with the EDE and BSS groups. In addition, the 0.01% CO group showed increased TBUT and decreased corneal staining score 7 days after the induction of desiccating stress compared to the EDE group. We also evaluated the inflammatory markers IFN-*γ* and IP-10 multiplex immunobead assay which indicated that eye drops containing CO extracts reduce inflammation of the ocular surface. Previous studies have confirmed that CO extracts exert anti-inflammatory effects by regulating the production of prostaglandin E_2_ and TNF-*α* gene expression through the COX-2 pathway [21].

Extensive literature review revealed that IFN-*γ* is the most frequently increased inflammation marker in the tears of patients with dry eye disease and that it plays a key role in the pathogenesis of dry eye and correlates with mechanisms of immune modulation; for example, it increases T-helper type 1 cells [43]. IP-10 is an IFN-*γ*/*α*- and TNF-*α*-inducible chemokine that is highly expressed by a variety of cells, including activated T lymphocytes, NK cells, and monocytes [44]. Our present findings support the results of recent studies showing that topical antioxidant also has anti-inflammatory activity in a murine dry eye model [6]. Furthermore, ROS levels detected using DCF-DA were notably lower in the 0.01% and 0.1% CO extract groups than in the EDE and BSS groups, suggesting that CO extracts protect the ocular surface from desiccating stress via antioxidative defense mechanisms in the murine dry eye model.

Eventually, we used an eye mask containing CO extracts to demonstrate the antioxidative effects in patients with dry eye disease. Both OSDI score and TBUT had significantly improved in the treatment group after 4 weeks of wearing the mask. In addition, significant differences between the two groups were observed in terms of OSDI score and TBUT. Taken together, these findings suggest that the masks containing CO extracts used in this study provide subjective and objective improvement in dry eye parameters, possibly via the antioxidative defense system.

Our findings are comparable to the results of previous studies that have demonstrated the efficacy of antioxidative and anti-inflammatory agents in improving dry eye parameters [3, 5, 16]. For instance, a mixture of four natural plant ethyl alcohol extracts decreased the levels of inflammatory cytokines and oxidative stress markers on the ocular surface in the murine dry eye model and antioxidant glasses containing extracts of medical plants which improved the OSDI score and TBUT in patients with dry eye disease [6, 16]. In addition, omega-3 polyunsaturated fatty acids, such as antioxidants, have been found to be effective in improving dry eye symptoms [45]. Furthermore, Kim et al. [46] reported that topical vitamin A eye drops contribute to improvements in blurred vision, TBUT, Schirmer's test, and findings from impression cytology in patients with dry eye disease.

In conclusion, CO extracts, especially at concentrations of 0.01% and 0.1%, may protect the ocular surface from desiccating stress and resultant oxidative stress. In addition, wearing an antioxidative mask containing CO extracts improved dry eye symptoms, as well as the clinical parameters of dry eye. CO extracts may be used as an adjunctive therapeutic option for the treatment of dry eye disease.

## Figures and Tables

**Table 1:** Ocular surface disease index scores in the treatment and placebo groups at baseline, 2 weeks, and 4 weeks after treatment.

	Follow-up periods			*P* value		
	Baseline	2 weeks	4 weeks	*P*1	*P*2	*P*3
Treatment group (*N* = 25)	25.20 ± 8.28	22.36 ± 10.10	18.20 ± 4.25	0.88	<0.01	<0.01
Placebo group (*N* = 25)	26.48 ± 9.21	25.84 ± 4.78	24.84 ± 3.42	0.99	0.79	0.85
*P* value	0.85	0.18	<0.01			

*P*1 baseline versus 2 weeks; *P*2 baseline versus4 weeks; and *P*3 2 weeks versus 4 weeks.

**Table 2:** Tear film break-up time in the treatment and placebo groups at baseline, 2 weeks, and 4 weeks after treatment.

	Follow-up periods			*P* value		
	Baseline	2 weeks	4 weeks	*P*1	*P*2	*P*3
Treatment group (*N* = 25)	7.12 ± 1.20	7.80 ± 1.12	8.79 ± 1.73	0.88	0.01	0.07
Placebo group (*N* = 25)	7.28 ± 1.46	7.36 ± 1.41	7.50 ± 1.47	0.96	0.91	0.94
*P* value	0.86	0.26	0.02			

*P*1 baseline versus 2 weeks; *P*2 baseline versus4 weeks; and *P*3 2 weeks versus 4 weeks.

**Table 3:** Schirmer's test value in the treatment and placebo groups at baseline, 2 weeks, and 4 weeks after treatment.

	Follow-up periods			*P* value		
	Baseline	2 weeks	4 weeks	*P*1	*P*2	*P*3
Treatment group (*N* = 25)	6.84 ± 1.03	6.92 ± 0.91	7.04 ± 1.49	0.99	0.98	0.98
Placebo group (*N* = 25)	6.56 ± 1.16	6.64 ± 1.08	6.91 ± 1.29	0.99	0.84	0.99
*P* value	0.50	0.42	0.87			

*P*1 baseline versus 2 weeks; *P*2 baseline versus4 weeks; and *P*3 2 weeks versus 4 weeks.
